# Global socioeconomic inequality in the burden of communicable and non-communicable diseases and injuries: an analysis on global burden of disease study 2019

**DOI:** 10.1186/s12889-021-11793-7

**Published:** 2021-09-28

**Authors:** Mehrnoosh Emadi, Sajad Delavari, Mohsen Bayati

**Affiliations:** Health Human Resources Research Center, School of Health Management and Information Sciences, Shiraz University of Medical Sciences, Almas Building, Alley 29, Qasrodasht Ave, Shiraz, Iran

**Keywords:** Inequality, Burden of diseases, Human development index, Communicable diseases, Non-communicable, Injuries

## Abstract

**Background:**

Examining the distribution of the burden of different communicable and non-communicable diseases and injuries worldwide can present proper evidence to global policymakers to deal with health inequality. The present study aimed to determine socioeconomic inequality in the burden of 25 groups of diseases between countries around the world in 2019.

**Methods:**

In the current study data according to 204 countries in the world was gathered from the Human Development Report and the Global Burden of Diseases study. Variables referring to incidence, prevalence, years of life lost (YLL), years lived with disability (YLD) and disability adjusted life years (DALY) resulting by 25 groups of diseases and injuries also human development index was applied for the analysis. For measurement of socioeconomic inequality, concentration index (CI) and curve was applied. CI is considered as one of the popular measures for inequality measurement. It ranges from − 1 to + 1. A positive value implies that a variable is concentrated among the higher socioeconomic status population and vice versa.

**Results:**

The findings showed that CI of the incidence, prevalence, YLL, YLD and DALY for all causes were − 0.0255, − 0.0035, − 0.1773, 0.0718 and − 0.0973, respectively. CI for total Communicable, Maternal, Neonatal, and Nutritional Diseases (CMNNDs) incidence, prevalence, YLL, YLD and DALY were estimated as − 0.0495, − 0.1355, − 0.5585, − 0.2801 and − 0.5203, respectively. Moreover, estimates indicated that CIs of incidence, prevalence, YLL, YLD and DALY for Non-Communicable Diseases (NCDs) were 0.1488, 0.1218, 0.1552, 0.1847 and 0.1669, respectively. Regarding injuries, the CIs of incidence, prevalence, YLL, YLD and DALY were determined as 0.0212, 0.1364, − 0.1605, 0.1146 and 0.3316, respectively. In the CMNNDs group, highest and lowest CI of DALY were related to the respiratory infections and tuberculosis (− 0.4291) and neglected tropical diseases and malaria (− 0.6872). Regarding NCDs, the highest and lowest CI for DALY is determined for neoplasms (0.3192) and other NCDs (− 0.0784). Moreover, the maximum and minimum of CI of DALY for injuries group were related to the transport injuries (0.0421) and unintentional injuries (− 0.0297).

**Conclusions:**

The distribution of all-causes and CMNNDs burden were more concentrated in low-HDI countries and there are pro-poor inequality. However, there is a pro-rich inequality for NCDs’ burden i.e. it was concentrated in high-HDI countries. On the other hand, the concentration of DALY, YLD, prevalence, and incidence in injuries was observed in the countries with higher HDI, while YLL was concentrated in low-HDI countries.

## Introduction

All health systems aim to restore, maintain, and improve community individuals’ health [1]. Health is considered a fundamental right and need for all humans [2], which justice is one of the dimensions of its establishment [3].

The concept of equity in health is embedded in a principle of human rights [4], which means a lack of systematic and potential differences in one or more fields of health among a population and socioeconomic subgroups [5]. The concept includes equity in health outcomes, financing and access to services [6, 7]. Equity in general is a subjective concept. However, for measuring it in practice inequality is emphasized. Inequality can be measured across different subgroups of population such as socioeconomic, gender, ethnicity, geography and etc. In this regards, socioeconomic inequality in health is a most common approaches. Socioeconomic inequalities in the health sector affect health indices in the whole community and deepen its poverty and inequality [8]. The inequalities may be a difference in health outcomes and accessing healthcare or getting disease [9] among populations with various socioeconomic status [10]. They are a specific type of difference in health in which more vulnerable social groups or those are facing adverse conditions and discrimination experience additional health risks and worse health systematically compared to those with desired social status, continuously [11]. The outcomes such as life expectancy, mortality rate, and disease burden can effectively reflect many health micro-factors’ resultant to assess justice in health status. The indicators can show health status and its inequality rate better and more comprehensive [4]. The indices related to the burden of different diseases can represent the diseases’ outcomes and create appropriate and more specific evidence for evaluating equity in health.

Based on the GBD study 2019, Communicable, Maternal, Neonatal, and Nutritional Diseases (CMNNDs) were responsible for 10.2 million (95% Uncertainty Interval (UI) 9·19–11·4) mortalities and 669 million (95% UI 593–758) DALYs. While the Non-Communicable Diseases (NCDs) led to 42 million (95% UI 40·1 to 43·9) mortalities and 1620 million (95% UI 1430 to 1820) DALYs. Besides, 4.30 million (95% UI 3·92–4·61) mortalities and 294 million (95% UI 226 to 275) DALYs occurred due to injuries. Further, CMNNDs, NCDs, and injuries were responsible for 18, 74.3, and 7.6% of all deaths in 2019, respectively [12].

Examining the distribution of the burden of different CMNNDs and NCDs and injuries worldwide can present proper evidence to global policymakers to deal with health inequality. Numerous studies have focused on equity in health and its other aspects, while no research assessed the whole indices of health outcomes in all diseases worldwide comprehensively. The present study aimed to determine socioeconomic inequality in the burden of diseases around the world in 2019.

## Methods

In the present cross-sectional study, the data of all countries in the world during 2019 were collected from two separate datasets including Human Development Index (HDI) [13] and burden of diseases data related to GBD study [14]. The former was used for socioeconomic status of the countries and the latter was used for health outcomes in countries. These datasets were explained later.

The GBD study is considered the most comprehensive epidemiological study worldwide, which provides an instrument for quantifying the health lost due to diseases, injuries, and risk factors. The Institute for Health Metrics and Evaluation (IHME), consisting of 3600 researchers from more than 145 countries, estimated the mortality and disability data related to more than 350 diseases and injuries in 204 countries and territories in terms of age and sex since 1990 until now [15].

The data related to all causes and 25 groups of first and second level of diseases were gathered based on grouping the causes of diseases in the GBD study. Diseases at level 1 include CMNNDs, NCDs and injuries. Moreover, at level 2 consist of HIV/AIDS, respiratory infections and tuberculosis, enteric infections, neglected tropical diseases and malaria, other infectious diseases, maternal and neonatal disorders, and nutritional deficiencies (in CMNNDs group); neoplasms, cardiovascular diseases, chronic respiratory diseases, digestive diseases, neurological disorders, mental disorders, substance use disorders, diabetes and kidney diseases, skin and subcutaneous diseases, sense organ diseases, musculoskeletal disorders, and other non-communicable diseases (in NCDs group); and transport injuries, unintentional injuries and self-harm and interpersonal violence (in injuries group) [16]. Therefore, we include three level 1 causes (CMNNDs, NCDs, and injuries) and 22 level 2 diseases (7 in CMNNDs, 12 in NCDs, and 3 in injuries).

Further, the burden of diseases for all-ages and both sexes in 2019 was extracted for each group using prevalence, incidence, DALY, years lived with disability (YLD) and the years of life lost (YLL) indices per 100,000 populations. The GBD data was obtained from the open database of the Global Burden of Disease 2019 Study in the GHDx [16].

Furthermore, HDI was applied to represent socioeconomic status, the numerical value of which varies between 0 and 1, and the value closer to 1 indicates more human development. HDI values were collected from the Human Development Report 2019, which was published by the United Nations Development Programme (Human Development Report 2019, 13). HDI considers the dimensions of a long and healthy life, knowledge, and living standards. More precisely, it is the geometric mean of life expectancy, education, and gross national product (GNP) per capita. In the human development report, countries are divided into those having very high (HDI equal or above 0.800), high (0.700–0.799), middle (0.550–0.699), and low (below 0.550) human development [13]. Human Development Reports calculate HDI for 195 countries in 2019. Therefore, analysis was limited to these 195 countries.

In the current study, descriptive statistics of diseases burden indices were assessed based on the HDI groups of countries. Then, the concentration index (CI) calculated for the prevalence, incidence, DALY, YLL, and YLD of total all causes, as well as 25 groups of the first and second level diseases was utilized for measuring socioeconomic inequality.

**Fig. 1:**
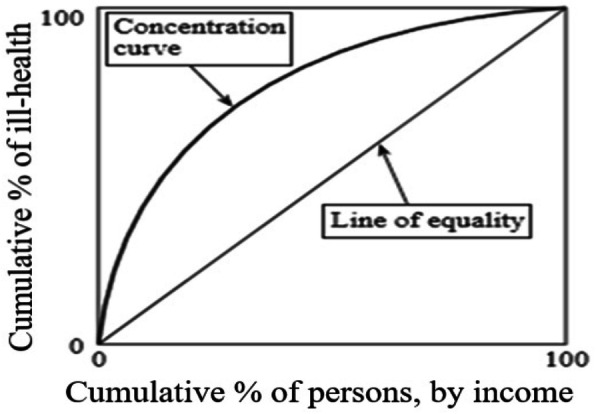
Concentration curve for health outcomes

CI is considered one of the most common methods for measuring inequality, developed based on the concentration curve (CC). The curve plots health variable as a cumulative percentage (y-axis) versus the cumulative percentage of the population ranked from the poorest to richest based on the economic status (x-axis). Besides, the curve follows the 45-degree line if all individuals possess the same health level, regardless of their economic status, which is called the equality line. However, CC is placed above the equality line when the health variable is more cumulated among the poor, which indicates the pro-poor inequality. The extent of inequality in health increases by distancing the curve from the equality line. CI is defined as twice the area enclosed by CC and 45-degree line and varies between + 1 and − 1. Thus, CI is equal to zero when the equality line and the curve coincide. Figure 1 displays the concept of CC and CI schematically [17].

Covariance approach was used for computing CI [17].

1 $$ C=\frac{2}{\mu}\mathit{\operatorname{cov}}\left( yi, Ri\right) $$

where C represents the concentration index, cov indicates covariance, and y refers to health outcome. Further, R and μ are considered the rank of country i in socioeconomic distribution and the mean health outcome.

If health variable has more concentration across the poor population, the CC lies above the equality line (CI < 0). Conversely, if health variable has more accumulation among the rich population, the CC is placed below the equality line (CI > 0). If health variable has complete equal distribution, the CC coincide with equality line (CI = 0) [18]. In the current study, negative values (curve above the 45-degree line) demonstrated the concentration of adverse health outcomes (disease burden) among low-HDI countries and vice versa.

Furthermore, the data were statistically analyzed by using STATA software version 14. For equity analysis in STATA we use Distributive Analysis Stata Package (DASP). Two main menus in the package including Inequality/ Gini and concentration indices and Curves/ Lorenz and concentration curves were used for estimations.

## Results

Table 1 summarizes the findings related to the distribution of the burden of different diseases among the various countries placed in HDI groups, which demonstrates the higher concentration of the burden of all diseases and CMNNDs ones in countries with less human development. However, the burden of NCDs and injuries is more concentrated in countries with greater HDI.

**Table 1:** Distribution of the burden of different diseases and injuries among countries by HDI group, 2019

**All causes**					
	DALY*	YLD*	YLL*	Prevalence*	Incidence*
Very high HDI	28,972.4 (27,320.6 to 30,624.3)	12,734.5 (12,320.2 to 13,148.7)	16,237.9 (14,759.7 to 17,716.2)	95,029.0 (94,754.2 to 95,303.8)	507,651.4 (500,819.7 to 514,483.1)
High HDI	30,256.1 (28,468.3 to 32,044.0)	10,864.6 (10,527.4 to 11,201.7)	19,391.5 (17,752.0 to 21,031.0)	95,624.0 (95,366.0 to 95,882)	527,602.7 (513,529.9 to 541,675.5)
Medium HDI	33,990.8 (31,357.5 to 36,624.0)	9574.8 (9316.3 to 9833.3)	24,415.9 (21,742.2 to 27,089.7)	95,935.4 (95,540.1 to 96,330.7)	544,758.5 (525,151.4 to 564,365.7)
Low HDI	47,804.3 (43,192.7 to 52,416.0)	9282.1 (9034.7 to 9529.5)	38,522.2 (33,934.3 to 43,110.0)	96,881.3 (96,531.8 to 97,230.8)	579,545.4 (565,585.6 to 593,505.4)
**Communicable, maternal, neonatal and nutritional (CMNN) diseases**					
	DALY*	YLD*	YLL*	Prevalence*	Incidence*
Very high HDI	1589.6 (1376.4 to 1802.9)	611.6 (565.3 to 658.0)	978.0 (802.4 to 1153.5)	36,711.7 (34,238.5 to 39,184.9)	313,876.8 (306,246.9 to 321,506.9)
High HDI	4642.0 (3410.4 to 5873.7)	1097.7 (973.3 to 1222.1)	3544.3 (2429.2 to 4659.4)	56,102.1 (53,281.1 to 58,923.2)	346,076.1 (333,611.8 to 358,540.5)
Medium HDI	11,643.7 (9014.6 to 14,272.8)	1643.5 (1436.4 to 1850.6)	10,000.2 (7547.1 to 12,453.3)	66,158.9 (63,143.3 to 69,174.5)	371,793.6 (353,400.3 to 390,186.9)
Low HDI	27,981.5 (23,661.3 to 23,661.3)	2418.4 (2231.1 to 2605.7)	25,563.1 (21,376.4 to 29,749.7)	74,339.7 (71,361.9 to 77,317.5)	403,093.8 (388,905 to 417,282.7)
**Non-communicable diseases (NCDs)**					
	DALY*	YLD*	YLL*	Prevalence*	Incidence*
Very high HDI	31,904.5 (28,161.1 to 35,647.9)	13,105.2 (11,765.4 to 14,445.0)	18,777.6 (16,144.9 to 21,410.3)	243,005.3 (230,633.4 to 255,377.2)	190,168.9 (162,503.7 to 217,834.2)
High HDI	23,782.0 (20,943.5 to 26,620.5)	8973.3 (8147.2 to 9799.4)	14,802.6 (12,655.6 to 16,949.5)	197,908.2 (186,619.7 to 209,196.8)	128,205.4 (107,245 to 149,165.9)
Medium HDI	19,271.5 (16,727.0 to 21,815.9)	7123.4 (6349.7 to 7897.2)	12,144.9 (10,217.5 to 14,072.3)	163,687.0 (150,307 to 177,067)	116,542.9 (93,725.2 to 139,360.6)
Low HDI	12,578.5 (11,746.6 to 13,410.3)	5134.1 (4937.4 to 5330.8)	7444.3 (6775.1 to 8113.5)	131,213.5 (127,433.4 to 134,993.7)	90,357.1 (87,486.2 to 93,228.0)
**Injuries**					
	DALY*	YLD*	YLL*	Prevalence*	Incidence*
Very high HDI	28,736.8 (20,059.2 to 37,414.4)	1069.9 (950.2 to 1189.6)	1385.8 (1157.7 to 1613.9)	32,117.0 (28,579.3 to 35,654.6)	8308.5 (6270.5 to 10,346.5)
High HDI	12,730.8 (6398.2 to 19,063.3)	805.8 (708.1 to 903.4)	2275.6 (1967.3 to 2583.8)	25,220.7 (22,238.2 to 28,203.2)	8173.9 (6814.2 to 9533.7)
Medium HDI	10,439.4 (4506.9 to 16,371.9)	635.2 (555.5 to 715.0)	2645.3 (2192.2 to 3098.4)	18,138.6 (15,979.3 to 20,298.0)	6450.0 (5339.3 to 7560.8)
Low HDI	3880.2 (3300.1 to 4460.3)	638.0 (510.6 to 765.3)	3242.2 (2711.6 to 3772.7)	16,854.7 (14,126.9 to 19,582.5)	7512.4 (6657.6 to 8367.2)

*All indices are presented as rate per 100,000 population. Figures in () show the 95% Uncertainty Interval (UI)

The study’s analytical results are provided in the form of CIs and CCs for the DALY, YLD, YLL, prevalence, and incidence of 25 groups of risk factors in CMNNDs and NCDs and injuries.

### All-cause, CMNNDs, NCD, and injuries

**Fig. 2:**
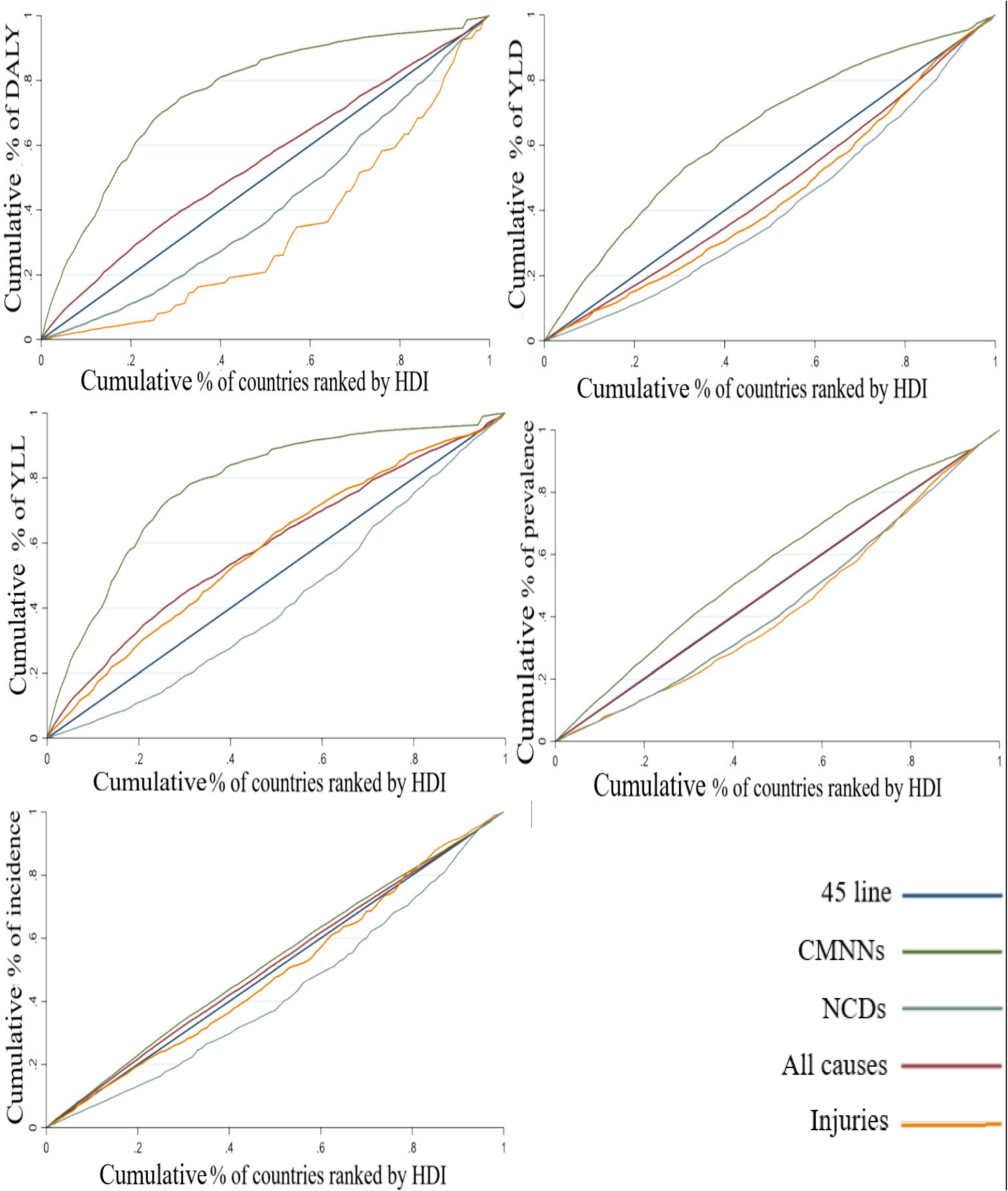
Concentration curves for the burden of all-cause, CMNN diseases, NCDs and injuries

Table 2 and Fig. 2 represent the data related to the CIs and CCs of DLAY, YLD, YLL, prevalence, and incidence in the groups of all-causes, CMNNDs, NCD, and injuries.

**Table 2:** Concentration index for all-causes, CMNN diseases, NCDs and injuries

Diseases	DALY*	YLD*	YLL*	Prevalence*	Incidence*
**All-cause**	**−0.0973 (−0.1258 to − 0.0687)**	**0.0718 (0.0629 to0.0808)**	**− 0.1773 (− 0.2171 to-0.1374)**	**−0.0035 (− 0.0045 to-0.0025)**	**−0.0255 (− 0.0326 to-0.0184)**
**Total CMNN diseases**	**− 0.5203 (− 0.5971 to-0.4436)**	**−0.2801 (− 0.3107 to-0.2495)**	**−0.5585 (− 0.6438 to-0.4731)**	**−0.1355 (− 0.1553 to-0.1157)**	**−0.0495 (− 0.0600 to-0.0389)**
HIV/AIDS	− 0.4365 (− 0.5293 to-0.3437)	−0.3578 (− 0.4554 to-0.2603)	−0.4434 (− 0.5360 to-0.3508)	−0.1272 (− 0.1674 to-0.0869)	−0.1575 (− 0.2028 to-0.1121)
Respiratory infections and Tuberculosis	− 0.4291 (− 0.5201 to-0.3381)	−0.4509 (− 0.5476 to-0.3542)	−0.1544 (− 0.1790 to-0.1298)	−0.0755 (− 0.1035 to-0.0475)	0.0036 (− 0.0095 to 0.0168)
Entric infections	−0.6089 (− 0.7031 to-0.5148)	−0.1446 (− 0.1770 to-0.1121)	−0.6799 (− 0.7846 to-0.5752)	−0.1386 (− 0.1709 to-0.1062)	−0.1338 (− 0.1649 to-0.1027)
Neglected Tropical Diseases and Malaria	− 0.6872 (− 0.7426 to-0.6317)	−0.5441 (− 0.6072 to-0.4810)	−0.7184 (− 0.7768 to-0.6600)	−0.4924 (− 0.5562 to-0.4285)	−0.6382 (− 0.7063 to-0.5702)
Other infectious diseases	− 0.5315 (− 0.7122 to-0.3508)	−0.2861 (− 0.3262 to-0.2459)	−0.5470 (− 0.7377 to-0.3563)	−0.2872 (− 0.3266 to-0.2477)	−0.2342 (− 0.2707 to-0.1977)
Maternal and neonatal disorders	− 0.4986 (− 0.5622 to-0.4350)	−0.1188 (− 0.1427 to-0.0949)	−0.5403 (− 0.6111 to-0.4694)	−0.1535 (− 0.1761 to-0.1309)	−0.2583 (− 0.2953 to-0.2213)
Nutritional deficiencies	− 0.4564 (− 0.5681 to-0.3446)	−0.3775 (− 0.4334 to-0.3216)	−0.5853 (− 0.7992 to-0.3714)	−0.2833 (− 0.3294 to-0.2372)	−0.1933 (− 0.2450 to-0.1416)
**Total NCDs**	**0.1669 (0.1355 to 0.1983)**	**0.1847 (0.1562 to 0.2133)**	**0.1552 (0.1192 to 0.1911)**	**0.1218 (0.1048 to 0.1388)**	**0.1488 (0.1099 to 0.1877)**
Neo plasma	0.3192 (0.2538 to 0.3845)	0.4544 (0.3492 to 0.5596)	0.2635 (0.2131 to 0.3139)	0.3461 (0.2571 to 0.4351)	0.3873 (0.2809 to 0.4937)
Cardiovascular diseases	0.0886 (0.0528 to 0.1243)	0.1290 (0.0982 to 0.1598)	0.0855 (0.0483 to 0.1227)	0.2112 (0.1818 to 0.2406)	0.3596 (0.2858 to 0.4335)
Chronic respiratory diseases	0.1987 (0.1355 to 0.2619)	0.1255 (0.0996 to 0.1513)	0.2162 (0.1409 to 0.2915)	0.1623 (0.1362 to 0.1884)	0.0505 (0.0306 to 0.0704)
Digestive diseases	−0.0113 (−0.0406 to 0.0180)	0.1998 (0.1618 to 0.2378)	−0.0694 (− 0.1033 to-0.0356)	−0.0034 (− 0.0340 to 0.0272)	0.0205 (− 0.0188 to 0.0599)
Neurological disorders	0.0767 (0.0533 to 0.1000)	0.0088 (− 0.0233 to 0.0410)	0.1755 (0.1357 to 0.2154)	0.0412 (0.0251 to 0.0574)	0.0133 (−0.0053 to 0.0320)
Mental disorders	0.0541 (0.0400 to 0.0683)	0.0171 (−0.0035 to 0.0378)	0.5160 (0.4156 to 0.6164)	0.1529 (0.1135 to 0.1922)	0.0967 (0.0660 to 0.1273)
Substance use disorders	0.2251 (0.1790 to 0.2713)	0.1823 (0.1455 to 0.2190)	0.3022 (0.2320 to 0.3724)	0.1737 (0.1376 to 0.2098)	0.1515 (0.1168 to 0.1862)
Diabetes and kidney diseases	0.0561 (0.0087 to 0.1036)	0.1807 (0.1395 to 0.2219)	−0.0185 (−0.0745 to 0.0375)	0.1733 (0.1437 to 0.2029)	0.1929 (0.1602 to 0.2256)
Skin and subcutaneous diseases	0.1313 (0.0900 to 0.1726)	0.1238 (0.0930 to 0.1546)	0.1629 (0.0416 to 0.2842)	0.1072 (0.0758 to 0.1387)	0.0125 (−0.0005 to 0.0255)
Sense organ diseases	0.0671 (0.0465 to 0.0876)	0.0627 (0.0416 to 0.0837)		0.0030 (−0.0461 to 0.0522)	0.4398 (0.3217 to 0.5578)
Musculoskeletal disorders	0.1676 (0.1435 to 0.1917)	0.1694 (0.1454 to 0.1934)	0.0524 (−0.0073 to 0.1121)	0.1515 (0.1248 to 0.1782)	0.1364 (0.1162 to 0.1566)
Other non-communicable diseases	−0.0784 (− 0.1178 to-0.0389)	0.1302 (0.0991 to 0.1614)	− 0.4088 (− 0.4691 to-0.3485)	−0.0571 (− 0.0795 to-0.0348)	−0.1112 (− 0.1484 to-0.0740)
**Injuries**	**0.3316 (0.2390 to 0.4243)**	**0.1146 (0.0779 to 0.1513)**	**−0.1605 (− 0.2062 to-0.1148)**	**0.1364 (0.1045 to 0.1683)**	**0.0212 (− 0.0347 to 0.0771)**
Transport injuries	0.0421 (−0.0110 to 0.0953)	0.2578 (0.1916 to 0.3241)	−0.0292 (− 0.0854 to 0.0269)	0.3416 (0.2645 to 0.4187)	0.2885 (0.2042 to 0.3728)
Unintentional injuries	−0.0297 (− 0.0678 to 0.0084)	0.1927 (0.1392 to 0.2463)	− 0.1530 (− 0.1957 to-0.1103)	0.2021 (0.1467 to 0.2574)	0.0726 (0.0208 to 0.1243)
Self-harm and interpersonal violence	−0.0231 (− 0.0819 to 0.0356)	0.1691 (0.0612 to 0.2770)	−0.0869 (− 0.1521 to-0.0217)	0.1690 (0.0887 to 0.2492)	0.3141 (0.2092 to 0.4189)

*Figures in () show the confidence intervals at 90% for CIs

Regarding all-causes, the CIs of DLAY, YLL, prevalence, and incidence were determined as negative, and the relevant curves were placed above the equality line by indicating the concentration of the diseases among lower HDI countries. However, the positive values were obtained for the CI of YLD, reflecting that this outcome is concentrated in the higher HDI countries in the world.

In total NCDs, CI was calculated as positive for all outcomes, and their CCs were below the equality line, which demonstrates the concentration of the total NCDs in countries with better socioeconomic status. However, CIs were negative in all outcomes of total CMNNDs diseases, and CCs were placed above the line, which means that CMNNDs are more concentrated in the countries having low socioeconomic status.

Additionally, the positive values were achieved for the CIs of DLAY, YLD, prevalence, and incidence in injuries by representing the concentration of injuries burden in the wealthy countries. In contrast, the negative values were observed in the CI of YLL, which indicates its concentration among poor ones.

### CMNNDs subgroups

**Fig. 3:**
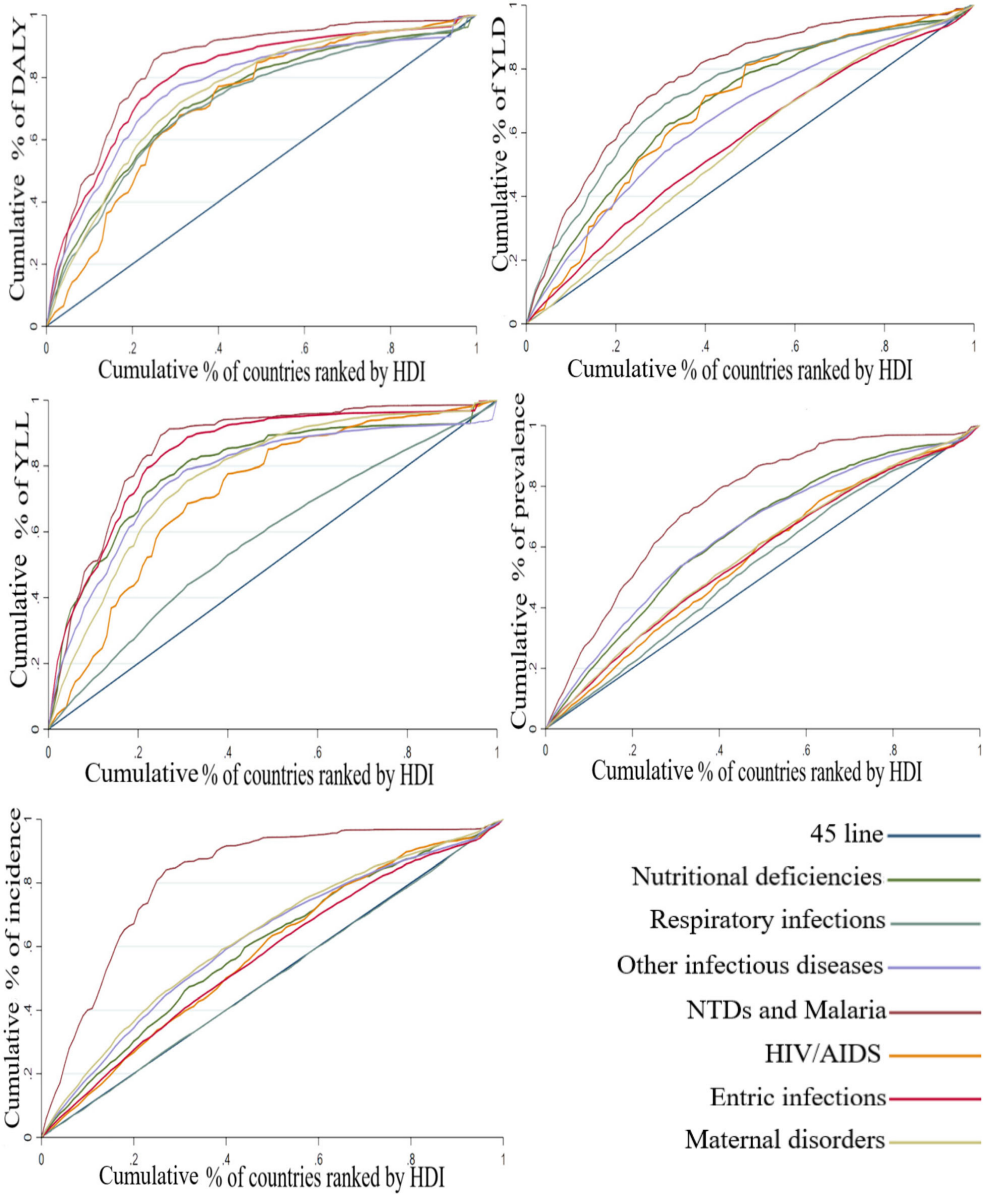
Concentration curves for the burden of CMNN diseases subgroups

The results regarding the CIs and CCs of DLAY, YLD, YLL, prevalence, and incidence in the different subgroups of CMNNDs are presented in Table 2 and Fig. 3. As shown, CI is negative for most of the diseases placed in the group of CMNNDs. Also, the maximum CI of DALY, YLL, prevalence, and incidence is obtained in respiratory infections and tuberculosis, while the minimum is determined for neglected tropical diseases (NTDs) and malaria. Further, the CI of YLD is maximized in maternal and neonatal disorders (− 0.118879) and minimized in NTDs and malaria (− 0.544148).

### NCDs subgroups

**Fig. 4:**
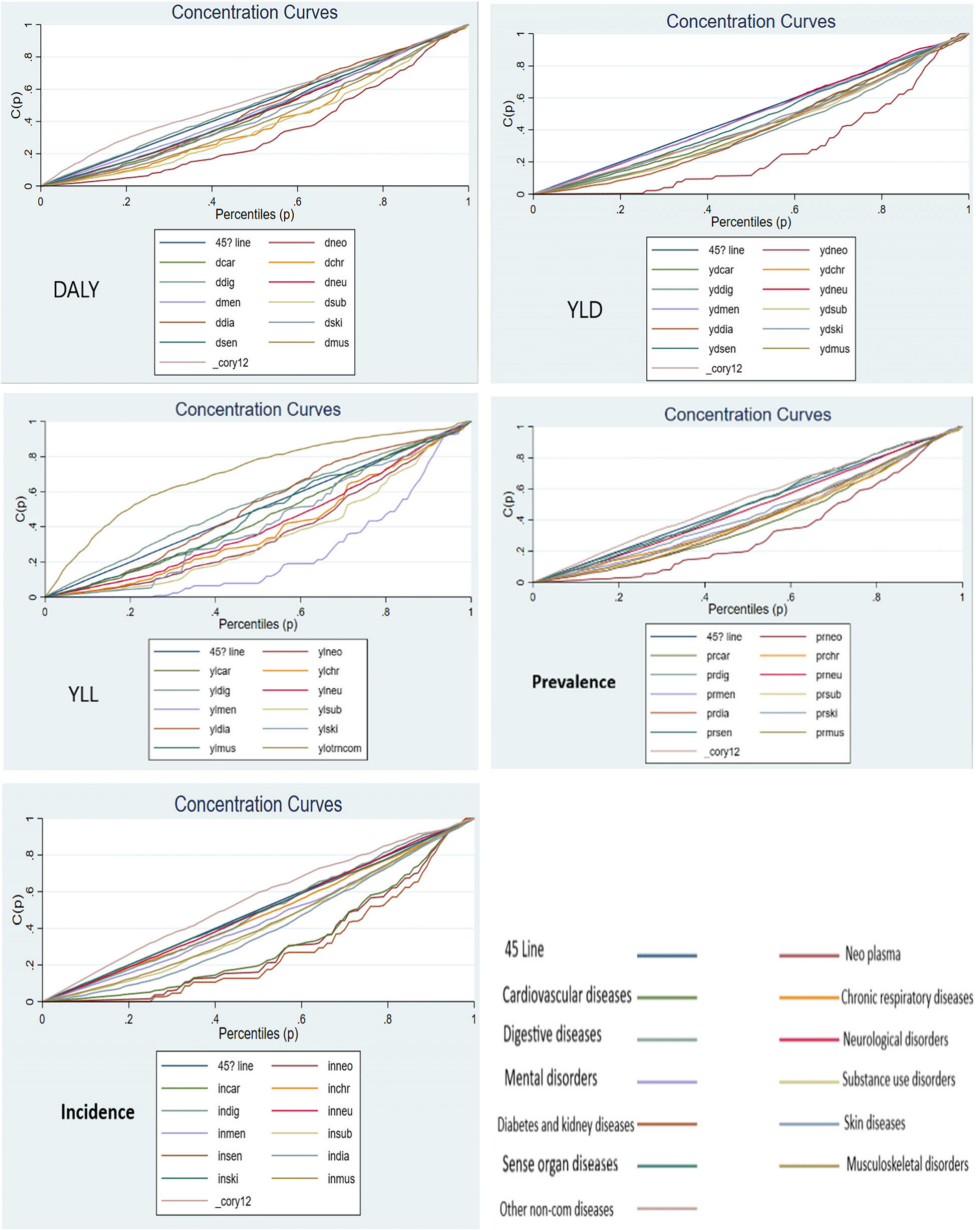
Concentration curves for the burden of NCDs subgroups

Based on the results related to the CIs and CCs of DLAY, YLD, YLL, prevalence, and incidence in the subgroups of NCDs (Table 2 and Fig. 4), positive CI was obtained for most of the diseases in the group. Additionally, the maximum CI of DALY and prevalence was related to neoplasms, while the minimum was determined in other NCDs, respectively. Regarding YLD, CI was respectively maximized and minimized in neoplasms (0.454434) and neurological disorders (0.008882). Further, mental disorders and other NCDs achieved the maximum (0.516033) and minimum CI of YLL (− 0.408846), respectively. Finally, the highest and least CIs of incidence were respectively observed in sense organ diseases (0.439825) and other NCDs (− 0.111250).

### Injuries subgroups

**Fig. 5:**
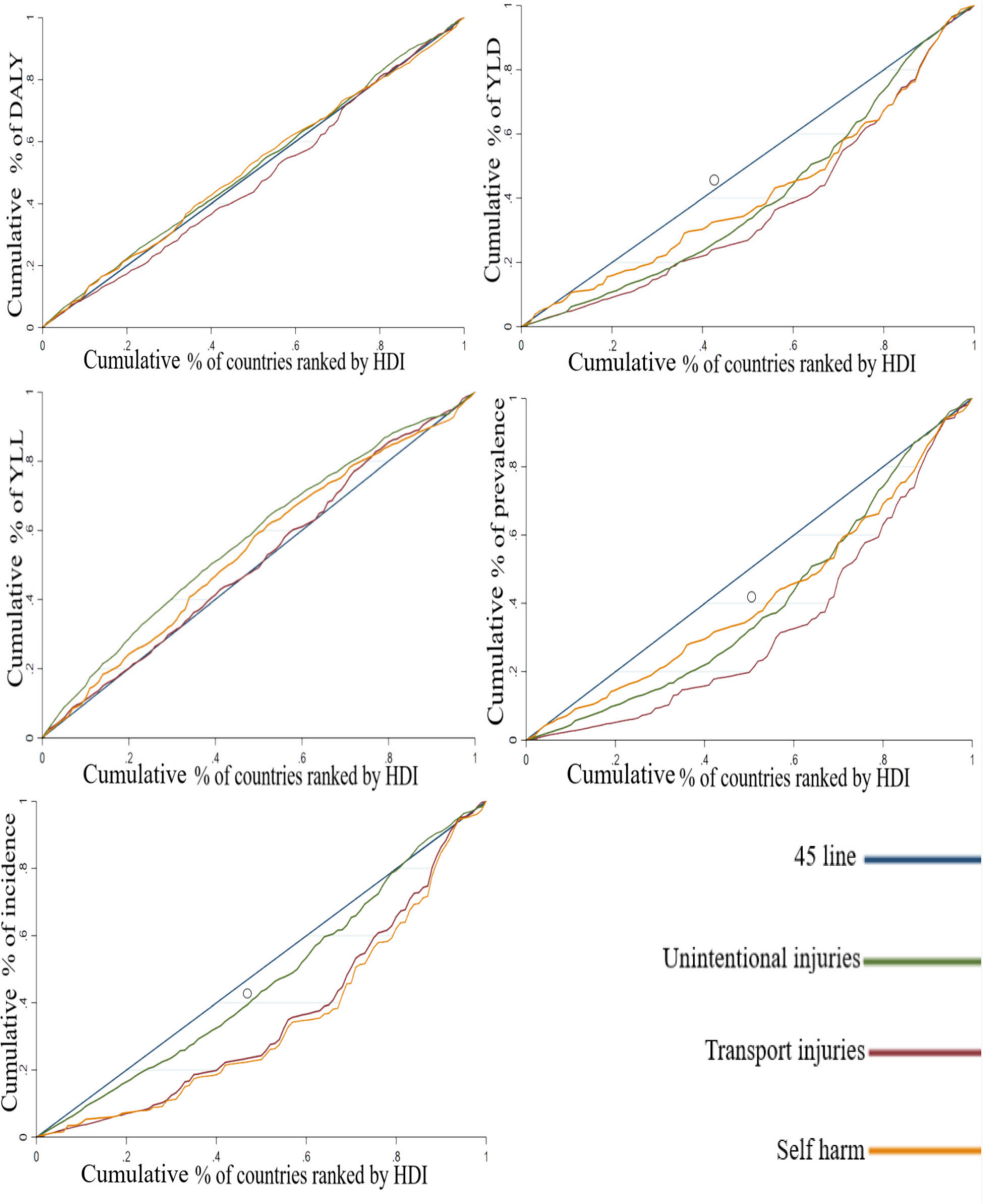
Concentration curves for the burden of injuries subgroups

Table 2 and Fig. 5 indicate the results of the CIs and CCs of DLAY, YLD, YLL, prevalence, and incidence for the subgroups of injuries. As shown, the maximum and minimum CIs of DALY and YLL are respectively determined for transport and unintentional injuries. Additionally, the highest CI of YLD and prevalence is obtained in transport injuries, while the least is observed in self-harm and interpersonal violence. Finally, the incidence rate is maximized in self-harm and interpersonal violence (0.314111) and minimized in unintentional injuries (0.072644).

## Discussion

Reducing health inequality in the world is always considered as one of the goals of global health policymakers. The present study was conducted to determine socioeconomic inequality in the burden of 25 groups of diseases among countries in the world in 2019.

Based on the study results, the burden of communicable and non- communicable diseases and injuries was unfairly distributed among countries worldwide. Considering the extent of the findings and the existence of limited similar evidences for all diseases especially at global level, only the diseases with similar studies were discussed. Findings according to each diseases group are discussed as follows.

The burden of total CMNNDs and most diseases in its group (neglected tropical diseases (NTDs), HIV/AIDS, nutritional deficiencies, enteric infections, maternal and neonatal disorders and other infectious diseases) was more concentrated in countries with lower HDI. So there are pro-poor inequality.

Hotez assessed 13 types of NTDs and found their higher prevalence among the individuals living in extreme poverty, especially in sub-Saharan Africa, Asia, Latin America, and the Caribbean [19]. Other studies reported a relationship between low socioeconomic status with the adverse health outcomes of NTDs [20–23]. NTDs are considered as a group of infectious diseases, which occur in equatorial and semi-tropical climates. They grow in environments where access to adequate sanitary facilities, clean water, and healthcare are limited. Individuals live adjacent to animals and infectious disease vectors such as remote and rural places, informal habitations, or affected regions. Therefore, the occurrence and complications of NTDs are directly related to socioeconomic condition [24].

According to Wabiri, the prevalence and indirect burden of HIV are higher among poor and vulnerable persons in South Africa [25]. In fact, the poor are more exposed to damage due to low awareness about HIV and low access to the required services such as HIV testing and treatment [25]. Wiswanath referred to low socioeconomic status persons’ tendency to have less information flow than their peers with a higher status [26]. Thus, inequalities in access to mass media follow inequalities in providing HIV services and marginalize poor and vulnerable individuals.

Emamian et al. found that nutritional deficiencies are greater among low socioeconomic status persons in Shahrod, and the group is more affected by the risk factor [27].

Low-income individuals less follow a healthy diet and prefer high-energy and low-nutritious foods compared to the rich [28, 29]. The diet of low-income groups is low energy, fiber, and vitamin [30]. Their health is at a greater risk concerning nutritional deficiencies due to economic barriers such as the lack of income and nutrition knowledge [31].

The results represented the higher concentration of total NCD’s burden and most diseases in its group (neoplasms, cardiovascular diseases, chronic respiratory diseases, neurological disorders, mental disorders, skin and subcutaneous diseases, substance use disorders and musculoskeletal disorders) among the countries with more HDI. So there are pro-rich inequality. The higher incidence and prevalence of NCDs in high income countries could be attributable to diagnostic equipments and procedures which are vastly available in these countries [32, 33]. Besides, countries with better socioeconomic status are more vulnerable to NCD risk factors such as smoking, low activity, unhealthy diet, air pollution, mental disorders, etc. therefore, higher burden of NCDs in countries with higher HDI could be justifiable according to literature [34–36].

The previous studies reported different results regarding the inequality of NCDs among other income communities. For example, the results of some research conducted in India and Bangladesh indicated the prevalence of NCDs in more developed cities, higher-income groups, and wealthier households [37–39]. The higher income is associated with growing the consumption of high-energy and unhealthy foods enriched in fat, sugar, and calorie, and increasing sedentary lifestyle, which can be considered one of the possible reasons for raising the prevalence of NCDs in high-income groups [24]. However, habitats in poor or marginalized communities have a higher risk of dying due to NCDs than the wealthy groups based on some studies’ results in other countries [40–42].

The findings indicated the higher concentration of neoplasms burden in the countries with higher HDI. In a research, it is reported that the rate of prevalence, incidence and premature death of top five cancers in 2018 was very higher in countries with more HDI [43]. The results of Soheilizad study in 2016 also revealed that the highest prevalence, incidence and death due to lung cancer occurred in countries with higher HDI [44]. More consumption of high-energy and unhealthy foods, and unhealthy lifestyles, could be a possible cause for higher burden of cancer in population with higher socioeconomic status [39].

Furthermore, a significant concentration was observed in all indices of cardiovascular disease (CVD) burden among high-HDI countries.

Based on the results of the different studies performed in India, the risk of CVD morbidity increased among groups with higher socioeconomic status [34, 45, 46]. Some of the possible reasons are their sedentary lifestyle and western food preferences which are related to urbanization [46]. However, other research reported possessing low socioeconomic status or living in low and middle-income countries as the causes of increasing CVD morbidity [24, 45]. In this regard, a study was conducted among ten European countries in 2006, which shows the higher rate of CVD mortality in the community with lower socioeconomic status [47].

The results of the present study demonstrated that the DALY, YLD, prevalence, and incidence indices of diabetes were significantly concentrated in high- HDI countries.

According to Corsi, the risk of diabetes morbidity is more among high-income groups in India [48]. Additionally, a positive relationship was reported between diabetes prevalence in the Dominican Republic with welfare [49], which can be related to unhealthy lifestyle among higher socioeconomic status groups [50]. Other studies referred to the greater risk of getting diabetes in obese [51] and sedentary individuals [52], and those with hypertension ones [53]. However, living in low- and middle-income countries enhances the risk of diabetes morbidity and bears a considerable burden of diseases and disability based on the studies conducted in Bangladesh, Iran, Turkey, and low-income countries [24, 37, 53–55].

Finally, the study results indicated the significant concentration of the YLD, prevalence, and incidence indices in injuries and all diseases in its group among high-HDI countries, as well as concentrating YLL index in low-HDI ones. Therefore, the mortality caused by the injuries is more concentrated in the lower developed countries despite their higher occurrence and prevalence in the more developed ones due to the lack of timely access to high-quality health services.

Burrows found that living in low-income countries is highly related to most causes of the mortalities caused by injury, especially fire, burns, and poisoning, by conducting a study in Canada [56]. The transport-related mortality among the boys aged 10–14 and 15–19 years old in the South Korean families with low income was more than twice compared to their peers in high-income families [57]. Individuals with greater socioeconomic status can possess wider resources to protect their health and safety (money, knowledge, credit, power, good social communications, and better roads in wealthier regions). In contrast, those with low socioeconomic status are devoid of such instruments [58, 59]. Further, the high cost of safety equipment is considered as one of the other barriers in the regions [60, 61].

The present study examined socioeconomic equity in all indicators of health outcomes (including incidence, prevalence, YLL, YLD, and DALY) in all diseases (25 group) comprehensively. As well, we estimate the CI for all groups independently and found that which diseases have pro-poor or pro-reach inequality. This is the main strength of the current research which could guide health policymakers across the globe. On the other hand, the ecological nature of the present study is one of its main limitations. Further micro-level studies are required to provide evidences for policy within each country, use in countries and special diseases despite performing the current study at macro and ecological level, and provide an overview regarding the between-country inequality of disease burden.

This study provide sound evidence about distribution of diseases’ burden. While this study has been performed at global level, other studies suggest that the same inequality could be seen also within the countries. Therefore, these findings could help policymakers at global and local level to decide about distribution of healthcare facilities and infrastructure improvement for different disease groups which varies in different locations.

## Conclusion

It is found that burden of for all-causes and CMNNDs were more concentrated in low-HDI countries and there are pro-poor inequality. However, there is a pro-rich inequality for NCDs’ burden i.e. it was concentrated in high-HDI countries. On the other hand, the concentration of DALY, YLD, prevalence, and incidence in injuries was observed in the countries with higher HDI, while YLL was concentrated in low-HDI countries.

## Data Availability

The data used in this study are publicly available in IHME (http://ghdx.healthdata.org/gbd-results-tool) and UN (http://hdr.undp.org/en/2019-report) databases.

## Abbreviations

CC: Concentration Curve
CI: Concentration Index
CMNNDs: Communicable, Maternal, Neonatal and Nutritional Diseases
DALY: Disability-Adjusted Life Years
GBD: Global Burden of Disease
GNP: Gross National Product
HDI: Human Development Index
IHME: Institute for Health Metrics and Evaluation
NCDs: Non-Communicable Diseases
NTDs: Neglected Tropical Diseases
UN: United Nations
YLD: Years Lived with Disability
YLL: The Years of Life Lost
